# Benzene Metabolite 1,2,4-Benzenetriol Induces Halogenated DNA and Tyrosines Representing Halogenative Stress in the HL-60 Human Myeloid Cell Line

**DOI:** 10.1289/ehp.1103437

**Published:** 2011-08-22

**Authors:** Takuro Nishikawa, Emiko Miyahara, Masahisa Horiuchi, Kimiko Izumo, Yasuhiro Okamoto, Yoshichika Kawai, Yoshifumi Kawano, Toru Takeuchi

**Affiliations:** 1 Department of Environmental Medicine, and; 2 Department of Pediatrics, Graduate School of Medical and Dental Sciences, Kagoshima University, Kagoshima, Japan; 3 Laboratory of Food and Biodynamics, Graduate School of Bioagricultural Sciences, Nagoya University, Nagoya, Japan

**Keywords:** benzene, hypochlorous acid, leukemia, myeloperoxidase, reactive oxygen species

## Abstract

Background: Although benzene is known to be myelotoxic and to cause myeloid leukemia in humans, the mechanism has not been elucidated.

Objectives: We focused on 1,2,4-benzenetriol (BT), a benzene metabolite that generates reactive oxygen species (ROS) by autoxidation, to investigate the toxicity of benzene leading to leukemogenesis.

Methods: After exposing HL-60 human myeloid cells to BT, we investigated the cellular effects, including apoptosis, ROS generation, DNA damage, and protein damage. We also investigated how the cellular effects of BT were modified by hydrogen peroxide (H_2_O_2_) scavenger catalase, hypochlorous acid (HOCl) scavenger methionine, and 4-aminobenzoic acid hydrazide (ABAH), a myeloperoxidase (MPO)-specific inhibitor.

Results: BT increased the levels of apoptosis and ROS, including superoxide (O_2_^•−^), H_2_O_2_, HOCl, and the hydroxyl radical (^•^OH). Catalase, ABAH, and methionine each inhibited the increased apoptosis caused by BT, and catalase and ABAH inhibited increases in HOCl and ^•^OH. Although BT exposure increased halogenated DNA, this increase was inhibited by catalase, methionine, and ABAH. BT exposure also increased the amount of halogenated tyrosines; however, it did not increase 8-oxo-deoxyguanosine.

Conclusions: We suggest that BT increases H_2_O_2_ intracellularly; this H_2_O_2_ is metabolized to HOCl by MPO, and this HOCl results in possibly cytotoxic binding of chlorine to DNA. Because myeloid cells copiously express MPO and because halogenated DNA may induce both genetic and epigenetic changes that contribute to carcinogenesis, halogenative stress may account for benzene-induced bone marrow disorders and myeloid leukemia.

Benzene, widely used in the chemical industry, is a common environmental contaminant found in gasoline, cigarette smoke, and coal tar. In humans, chronic exposure to benzene results in progressive deterioration in hematopoiesis, possibly leading to myelodysplastic syndrome and acute myeloid leukemia (Aksoy 1989; Huff 2007).

Although the mechanisms of benzene toxicity remain unclear, it is considered to occur only after metabolic activation (Snyder and Hedli 1996; Whysner et al. 2004). In the liver, benzene is primarily metabolized by cytochrome P450 2E1 (CYP2E1) to benzene oxide, which is then converted by epoxide hydrolase to dihydrodiol. Subsequent processing by dihydrodiol dehydrogenase yields catechol (CT). Alternatively, by nonenzymatic rearrangement, benzene oxide is converted to phenol, which can be oxidized by CYP2E1 to 1,4-hydroquinone (HQ) and 1,4-benzoquinone (Snyder and Hedli 1996). The pathway for formation of 1,2,4-benzenetriol (BT) in humans is not yet clearly understood; it has been suggested that BT may be formed by the hydroxylation of either HQ or CT (Henderson et al. 1989; Inoue et al. 1989).

Various metabolites of benzene are considered to bring out toxicity through the generation of reactive oxygen species (ROS), inhibition of topoisomerase, and subsequent induction of DNA damage (Whysner et al. 2004). Among benzene metabolites, the triphenolic metabolite BT reacts most actively with molecular oxygen (Lewis et al. 1988; Zhang et al. 1996). We also know that BT induces oxidative DNA damage and breaks DNA strands (Kawanishi et al. 1989; Kolachana et al. 1993; Lewis et al. 1988). Moreover, BT damages DNA more severely than does HQ, and benzene and CT per se have no detectable effects on DNA (Kawanishi et al. 1989). Because epidemiological studies of HQ and 1,4-benzoquinone have never demonstrated carcinogenicity in humans, the International Agency for Research on Cancer (IARC) assigned their carcinogenic risk to humans as group 3: not classifiable as to carcinogenicity to humans (IARC 2011). Meanwhile, IARC has not evaluated the carcinogenicity of BT.

The heme enzyme myeloperoxidase (MPO), which is synthesized and secreted by neutrophils, monocytes, and other myeloid cells, is an important source of oxidants. MPO catalyzes the formation of hypochlorous acid (HOCl), a powerful oxidant derived from chloride ions and hydrogen peroxide (H_2_O_2_). HOCl is a potent cytotoxin that plays key roles in host defense by oxidizing the cellular constituents of invading pathogens (Hurst and Barrette 1989). At the same time, HOCl is also capable of damaging proteins, lipids, and nucleic acids in host tissue (Heller et al. 2000). By damaging the DNA of host cells, MPO-induced DNA halogenation might contribute to the association between chronic inflammation and cancer (Marnett 2000).

Although benzene is known to be specifically toxic to bone marrow in humans, the mechanism for this is not understood (Whysner et al. 2004). MPO has a much higher endogenous presence in bone marrow than in any other internal organ (Heller et al. 2000), but no previous study has examined the role of MPO-derived HOCl in benzene toxicity.

We investigated the effect of MPO-derived HOCl on the toxicity of BT in the HL-60 human myeloid cell line. To examine DNA damage induced by BT, we used an immunocytometric method to evaluate halogenated DNA, and we determined 8-oxo-deoxyguanosine (8-oxo-dG) levels using high-performance liquid chromatography (HPLC) coupled with electrochemical detection (ECD). We found that BT generates HOCl via the H_2_O_2_–MPO–halide system; rather than generating 8-oxo-dG, this HOCl halogenates DNA.

## Materials and Methods

*Cell culture.* The HL-60 human promyelocytic leukemia cell line was kindly supplied by the Japanese Cancer Research Resource Bank (Osaka, Japan). Cells were maintained in RPMI 1640 medium (Sigma-Aldrich, St. Louis, MO, USA) containing 10% heat-inactivated fetal calf serum (FCS; Hyclone, Logan, UT, USA) at 37°C in a humidified atmosphere with 5% carbon dioxide (CO_2_).

*Reagents.* We purchased BT, HQ, and CT from Wako Pure Chemical Industries (Osaka, Japan); catalase from Boehringer-Mannheim (Mannheim, Germany); 4-aminobenzoic acid hydrazide (ABAH) from Tokyo Chemical Industry (Tokyo, Japan); and methionine and sodium hypochlorite (NaOCl) from Nacalai Tesque (Kyoto, Japan).

*Determination of apoptosis by flow cytometry.* We used annexin V–fluorescein isothiocyanate (FITC) and propidium iodide (PI) double-labeling kits (TACS Annexin V-FITC Kit; Trevigen, Gaithersburg, MD, USA) to detect phosphatidylserine as a marker of apoptosis. HL-60 cells suspended in RPMI 1640/10% FCS at 4 × 10^5^/mL were exposed to BT (25–100 µM) at 37°C in 5% CO_2_ for 8 hr. For experiments with catalase (H_2_O_2_ scavenger), cells were exposed to BT plus 250 U/mL catalase. For experiments with ABAH (MPO inhibitor) and methionine (HOCl scavenger), HL-60 cells were preincubated with RPMI 1640/10% FCS containing 100 μM ABAH or 25 mM methionine for 24 hr; media was then replaced with new media containing the reagent plus BT. Unexposed HL-60 cells were used as controls. After incubation, cells were harvested and washed and then stained with annexin V–FITC and PI according to the manufacturer’s instructions. We evaluated the cells using a FACScan flow cytometer (Becton Dickinson, Mountain View, CA, USA), and data were analyzed using WinMDI software (version 2.9; Biology Software Net, La Habra, CA, USA). *Determination of intracellular ROS generation by flow cytometry.* To detect intracellular superoxide (O_2_^•−^) and H_2_O_2_, we followed the method of Takeuchi et al. (1996). Briefly, HL-60 cells suspended in phenol-red–free RPMI 1640 at 4 × 10^5^/mL were incubated with hydroethidine (HE; Molecular Probes, Carlsbad, CA, USA) or dichlorofluorescin-diacetate (DCFH-DA; Molecular Probes). The probe-loaded cells were then exposed to BT with or without 250 U/mL catalase for 30 min at 37°C. For experiments with ABAH or methionine, the cells were pretreated as described above and then suspended in phenol-red–free RPMI 1640 at 4 × 10^5^/mL. After addition of the same concentration of ABAH or methionine, loaded with probes, the cells were exposed to BT for 30 min at 37°C. Nonfluorescent HE is oxidized to fluorescent 2-hydroxyethidium by O_2_^•−^, whereas DCFH is oxidized to dichlorofluorescein (DCF) by H_2_O_2_ and peroxidases (Rothe and Valet 1990). Presence of 2-hydroxyethidium or DCF was measured by FACScan.

For selective detection of HOCl and the hydroxyl radical (^•^OH), HL-60 cells suspended in phenol-red-free–RPMI 1640 at 4 × 10^5^/mL were incubated with 10 μM aminophenyl fluorescein (APF; Sekisui Medical, Tokyo, Japan) or 10 μM hydroxyphenyl fluorescein (HPF; Sekisui Medical) and then exposed to BT for 30 min at 37°C with or without 250 U/mL catalase. For experiments with ABAH or methionine, cells were pretreated and exposed as described above. APF and HPF themselves are not highly fluorescent, but when reacted with HOCl (APF) or ^•^OH (HPF) they exhibit strong dose-dependent fluorescence, which can be used to differentiate HOCl and ^•^OH from H_2_O_2_, nitric oxide, and O_2_^•−^ (Setsukinai et al. 2003). The specificity and usefulness of these probes have been described previously (Kohanski et al. 2007; Nakazato et al. 2007). We measured the fluorescence intensity of cells by FACScan and analyzed data using WinMDI software.

*Determination of halogenated DNA by immunocytometric analysis.* To detect DNA damage by HOCl, we analyzed halogenated DNA using a novel monoclonal antibody (mAb2D3) that recognizes the HOCl-modified 2´-deoxycytidine residue 5-chloro-2´-deoxycytidine (5-CldC; supplied by Y. Kawai, Nagoya University, Nagoya, Japan) (Kawai et al. 2004, 2008). HL-60 cells were suspended in RPMI 1640/10% FCS at 1 × 10^6^/mL and then exposed to 50 μM BT with or without catalase. For experiments with ABAH or methionine, cells were pretreated as described above and then exposed to BT for 1 hr or 4 hr at 37°C in 5% CO_2_. HL-60 cells were exposed to 20 μM HQ or 1 mM NaOCl for 1 hr or 4 hr at 37°C in 5% CO_2_. After exposure, the cells were washed with phosphate-buffered saline (PBS) and then fixed in 4% paraformaldehyde (Wako Pure Chemical) at 4°C for 20 hr. We evaluated halogenated DNA as described elsewhere (Kawai et al. 2004, 2008), with minor modifications. Briefly, the fixed cells were permeabilized by a 3-min exposure, on ice, to PBS containing 0.3% Triton X-100. The cells were then blocked with 2% bovine serum albumin (Sigma-Aldrich) in PBS containing 0.05% Tween 20 (TPBS). The cells were then incubated with mAb2D3 in TPBS for 1 hr at room temperature. After washing with TPBS, the cells were incubated in TPBS for 1 hr at room temperature with FITC-labeled anti-mouse IgG (Dako, Kyoto, Japan). After incubation, cells were washed with TPBS and their fluorescence intensity was measured by FACScan. The data were analyzed as described above.

*Determination of 8-oxo-dG by HPLC-ECD.* To detect oxidative DNA damage by ^•^OH, we evaluated 8-oxo-dG by HPLC-ECD. Cells were suspended in RPMI 1640/10% FCS at 1 × 10^6^/mL and exposed to 50 μM BT for 1, 2, or 4 hr; 20 μM HQ for 1 or 4 hr; or 20 μM CT for 2 hr, with all exposures at 37°C in 5% CO_2_. The cells were immediately chilled in an ice-water bath, washed with ice-cold PBS, and then stored for later analysis as cell pellets at –80°C. DNA was extracted from the cells with DNA Extractor WB Kit (Wako Pure Chemical) according to the manufacturer’s instructions and enzymatically digested to nucleosides, as described by Takeuchi et al. (1994). After HPLC separation, 8-oxo-dG was detected by ECD, and deoxyguanosine (dG) was detected by ultraviolet absorption as described elsewhere (Takeuchi et al. 1994). 8-oxo-dG level was expressed as the molar ratio of 8-oxo-dG per 10^5^ dG.

*Immunocytochemical detection of halogenated tyrosines.* To detect protein damage by HOCl, we analyzed halogenated tyrosines using rabbit anti-chlorotyrosine antibody (Hycult Biotech, Uden, the Netherlands) (Gujral et al. 2003) and mouse anti-dibromotyrosine monoclonal antibody (JaiCA, Shizuoka, Japan), which cross-reacts with dichlorotyrosine (Kato et al. 2005). Cells were suspended in RPMI 1640/10% FCS at 4 × 10^5^/mL and then exposed to 50 μM BT at 37°C in 5% CO_2_ for 4 hr. After exposure, the cells were washed with PBS and centrifuged with Shandon Cytospin 4 (Thermo Scientific, Kanagawa, Japan) at 1,000 rpm for 8 min. Centrifuged cells on slides were dried and fixed with cold acetone and then blocked with PBS containing 2% bovine serum albumin. To detect chlorotyrosine, cells were incubated with anti-chlorotyrosine antibody and then stained with Alexa Fluor 488–conjugated goat anti-rabbit antibody (Invitrogen, Tokyo, Japan) and 1 μg/mL PI. To detect dibromo/dichlorotyrosine, cells were incubated with anti-dibromotyrosine antibody and then stained with Alexa Fluor 488–conjugated goat anti-mouse antibody and 1 μg/mL PI. The stained slides were examined by fluorescence microscopy.

*Statistical analysis.* Data are presented as mean + SE. Statistical analyses were performed using PASW Statistics software (version 18.0; SPSS, Inc., Tokyo, Japan). Treatment effects were established by nonparametric Wilcoxon tests. Data for DNA damage were analyzed using analysis of variance, followed by Fisher’s protected least significant difference test for post hoc comparisons of individual treatments. *p*-Values < 0.05 (two tailed) were considered significant.

## Results

**Figure 1:**
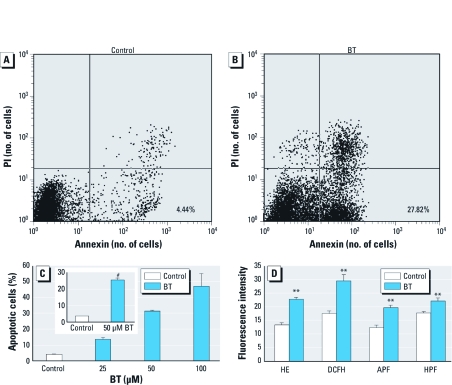
Apoptosis and intracellular ROS. (*A*,*B*) Representative dot graphs of unexposed HL-60 cells (control; *A*) and cells exposed to 50 μM BT for 8 hr (*B*). Cells in the lower right quadrant, which were stained with annexin V but not PI, were considered to be apoptotic; percentages of these cells in are shown in the figure. (*C*) Percentages of apoptotic HL-60 cells after exposure to BT for 8 hr; data presented are mean + SE from two independent experiments conducted in duplicate. Inset, percentages of apoptotic cells in controls or HL-60 cells exposed to 50 μM BT for 8 hr (mean + SE of 15 independent experiments conducted in duplicate). (*D*) Fluorescence intensities, corresponding to levels of various ROS, in controls or cells exposed to 50 μM BT for 30 min (mean + SE from 5–7 independent experiments conducted in duplicate). Fluorescence intensity is shown in arbitrary units. **p < 0.01, and ^#^*p* < 0.001, compared with control.

*Levels of apoptosis and intracellular ROS after BT exposure.* We found more annexin V–positive and PI-negative cells, considered to be apoptotic, in HL-60 cells that had been exposed to 50 µM BT for 8 hr (Figure 1B) than in controls (Figure 1A). The percentage of apoptotic cells in HL-60 cells exposed to 50 μM BT was significantly greater than in unexposed cells (Figure 1C, inset). Apoptosis increased depending on the concentration of BT (Figure 1C).

We determined intracellular ROS flow cytometrically using ROS-sensitive fluorescent probes: HE for O_2_^•−^, DCFH-DA for H_2_O_2_, APF for HOCl, and HPF for ^•^OH. BT increased the intracellular levels of each of these ROS (Figure 1D).

**Figure 2:**
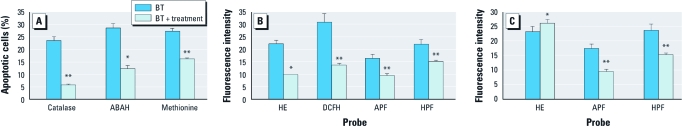
Suppression of BT-induced apoptosis and ROS generation by ROS scavengers (catalase and methionine) and an MPO inhibitor (ABAH). (*A*) Percentages of apoptotic cells in HL-60 exposed to 50 μM BT for 8 hr in the presence of MPO inhibitor and ROS scavengers; data presented are mean +SE from four to six independent experiments conducted in duplicate. (*B*) Effects of catalase on ROS generated by BT, as shown by fluorescence intensity of HE (for O_2_^•−^), DCFH‑DA (for H_2_O_2_), APF (for HOCl), and HPF (for ^•^OH) in cells exposed to BT or BT plus catalase for 30 min (mean + SE from three to five independent experiments conducted in duplicate). (*C*) Effects of ABAH on ROS generated by BT, as shown by fluorescence intensity of HE, APF, and HPF in cells exposed for 30 min to BT or BT plus ABAH (mean + SE from three to five independent experiments conducted in duplicate). *p < 0.05, and **p < 0.01 compared with the corresponding cells exposed to BT alone.

*Effect of ROS scavengers and MPO inhibitor on apoptosis and intracellular ROS.* Apoptosis was inhibited by catalase, ABAH, and methionine (Figure 2A). Catalase also inhibited the generation of O_2_^•−^, H_2_O_2_, HOCl, and ^•^OH induced by BT exposure (Figure 2B). Although ABAH inhibited the BT-induced increase of HOCl and ^•^OH, it further increased the generation of O_2_^•−^ (Figure 2C). We could not determine the effect of ABAH on H_2_O_2_ because peroxidases, which are required for the conversion of DCFH to DCF, were inhibited by ABAH (Matsugo et al. 2006).

**Figure 3:**
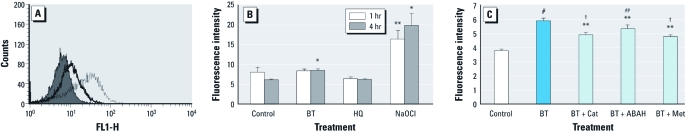
Induction of halogenated DNA in HL-60 cells by BT. (*A*) Histograms showing results for control HL-60 cells (gray shaded area) and cells exposed for 4 hr to 50 μM BT (black line) or 1 mM NaOCl (gray dotted line). FL1-H, height of green fluorescence. (*B*) Fluorescence intensities (arbitrary units) of controls or cells exposed to 50 μM BT, 20 μM HQ, or 1 mM NaOCl for 1 or 4 hr; data presented are mean + SE from 4 independent experiments conducted in duplicate. (*C*) Fluorescence intensities of controls or cells exposed for 4 hr to 50 μM BT, 50 μM BT with catalase (BT+Cat), 50 μM BT with ABAH (BT+ABAH), or 50 μM BT with methionine (BT+Met) (mean + SE of 3 independent experiments conducted in duplicate). *p < 0.05 compared with the corresponding control. **p < 0.01, and ^#^*p* < 0.001 compared with control. ^##^*p* < 0.05, ^†^*p* < 0.01 compared with the corresponding HL-60 cells that were exposed to BT alone.

*Levels of halogenated DNA.* Using flow cytometry after immunostaining, we measured the level of halogenated DNA in HL-60 cells exposed to 50 μM BT. HL-60 cells exposed to BT for 1 hr showed about the same levels of halogenated DNA as control (unexposed) cells. However, after 4 hr exposure to BT, increased levels of halogenated DNA were apparent (Figure 3A,B). Although catalase, methionine, and ABAH inhibited these increases (Figure 3C), the levels of halogenated DNA were still higher than those in control cells. HL-60 cells exposed to 1 mM NaOCl for 1 hr and for 4 hr had significantly more halogenated DNA (Figure 3B). In contrast, exposure to HQ did not increase the level of halogenated DNA (Figure 3B).

**Figure 4:**
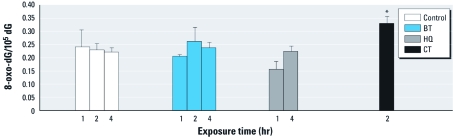
8-oxo-dG levels (expressed as the molar ratio of 8-oxo-dG per 10^5^ dG) in control HL-60 cells or cells exposed to 50 μM BT, 20 μM HQ, or 20 μM CT for 1, 2, or 4 hr; data presented are mean + SE of four independent experiments. *p < 0.05 compared with in corresponding control.

*Levels of 8-oxo-dG.* Using HPLC-ECD, we measured 8-oxo-dG levels in HL-60 cells exposed to 50 μM BT, 20 μM HQ, or 20 μM CT. HL-60 cells exposed to 20 μM CT for 2 hr had significantly more 8-oxo-dG. However, BT and HQ had about the same 8-oxo-dG levels as the control cells (Figure 4).

**Figure 5:**
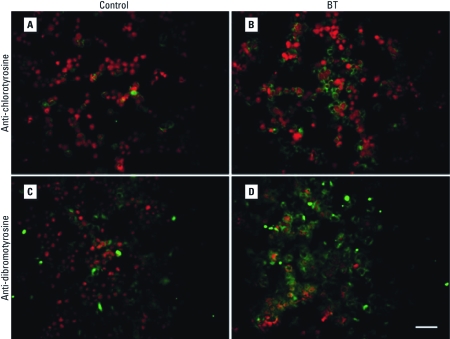
Halogenated tyrosines in unexposed (control) HL-60 cells (*A*, *C*) and cells exposed to 50 μM BT (*B*, *D*) stained with anti-chlorotyrosine antibody (*A*, *B*) or anti-dibromotyrosine antibody (*C*, *D*). See “Materials and Methods” for details. Green indicates halogenated tyrosines stained with Alexa Fluor 488; red indicates nucleic acids stained with PI. Magnification, 200×. Bar = 50 μm.

*Detection of halogenated tyrosines.* To confirm the induction of halogenative stress in HL-60 cells by BT, we detected HOCl-induced protein damage in the form of halogenated tyrosines. After 4 hr exposure to 50 μM BT, levels of both chlorotyrosine (Figure 5A,B) and dibromo/dichlorotyrosine (Figure 5C,D) were elevated.

## Discussion

Because the findings of *in vivo* and *in vitro* research so strongly implicate the involvement of ROS in benzene-induced toxicity (Snyder and Hedli 1996), we designed a study to investigate the carcinogenic mechanism of benzene, focusing on BT, a benzene metabolite that generates ROS by autoxidation (Kawanishi et al. 1989; Zhang et al. 1996). We specifically examined the cytotoxic effects of BT on a human myeloid cell line, a class of cells from the organ mainly affected by benzene. BT induced apoptosis in HL-60 cells in a concentration-dependent manner. Several ROS, including O_2_^•−^, H_2_O_2_, HOCl, and ^•^OH, were generated by exposure to 50 μM BT. The significant inhibition of BT-induced apoptosis in the presence of methionine, a relatively specific scavenger of HOCl (Tomono et al. 2009), suggests that BT generates HOCl. Moreover, BT exposure increased the amount of halogenated DNA and halogenated tyrosines detected by immunological examinations, which confirms generation of HOCl by BT. To the best of our knowledge, no previous studies have evaluated the generation of HOCl by benzene and its metabolites.

To investigate the mechanism of MPO-mediated apoptosis in HL-60 cells, we co-incubated HL-60 cells with BT and catalase or pretreated cells with ABAH or methionine, and then exposed them to BT. These reagents drastically suppressed the level of BT-induced apoptosis. This strongly implicates the H_2_O_2_–MPO–HOCl system in the induction of apoptosis by BT. Catalase inhibited BT-induced generation of ROS, and ABAH specifically inhibited BT-induced generation of HOCl and ^•^OH. This inhibition indicates that HOCl was generated via the H_2_O_2_–MPO–HOCl system. The inhibition of HOCl generation by both catalase and ABAH demonstrates that, in HL-60 cells exposed to BT, H_2_O_2_ was certainly metabolized to HOCl by MPO. It also suggests that HOCl might trigger BT-induced apoptosis of HL-60 cells. In contrast, ABAH further increased the BT-induced generation of O_2_^•−^, which indicates an accumulation of O_2_^•−^ caused by the inhibition of MPO and also indicates that O_2_^•−^ and probably H_2_O_2_ do not directly trigger apoptosis.

We then investigated whether this cytotoxicity of BT was related to the induction of DNA damage. We evaluated the halogenation of DNA by HOCl, and 8-oxo-dG induction by ^•^OH. Although CT exposure increased 8-oxo-dG as previously reported (Oikawa et al. 2001), BT exposure did not. In HL-60 cells exposed to BT, however, we did detect more halogenated DNA. Furthermore, catalase, ABAH, and methionine clearly inhibited DNA halogenation. These findings indicate that HOCl was generated by MPO after BT exposure, and that this HOCl was the likely culprit in DNA halogenation. We also tested whether HQ induces DNA damage. However, 20 μM HQ did not increase either the halogenated DNA or 8-oxo-dG. These results may help explain why HQ does not induce leukemia in humans (Levitt 2007). HQ is known to autoxidize more slowly and simply than does BT (Kawanishi et al. 1989), possibly accounting for the difference between BT and HQ; further study is required to confirm this.

Among halogenated nucleosides resulting from reaction with HOCl, 5-CldC, 5-chlorouracil, 8-chloro-deoxyadenine, and 8-chlorodeoxyguanine have been identified (Whiteman et al. 1997), with 5-CldC being the predominant carbon-chlorinated nucleoside product (Henderson et al. 1999). The mAb2D3 monoclonal antibody that we used in this experiment recognizes mainly 5-CldC (Kawai et al. 2004, 2008). Exposing DNA to HOCl causes large increases in pyrimidine oxidation, with no evidence of purine oxidation (i.e., 8-oxo-dG) (Whiteman et al. 1997). This effect is consistent with our finding of halogen-​damaged DNA, such as 5-CldC, and no evidence of increased 8-oxo-dG. In contrast, exposing DNA to HOCl has been reported to increase the 8-oxo-dG level (Ohnishi et al. 2002); however, that finding may have been due to the addition of diethylenetriaminepentaacetic acid to the reaction mixture. In another study, Kolachana et al. (1993) reported the induction of 8-oxo-dG in HL-60 cells by BT. The discrepancy between our results and those of that study may be explained by the difference in the precision of HPLC-ECD methods. In unexposed cells, we detected levels of 8-oxo-dG about 0.2 per 10^5^ dG. By contrast, assuming that the average molecular weight of nucleotides is 300, the 8-oxo-dG level in the other report was about 9.6 per 10^5^ dG.

Oxidative DNA damage has been implicated in carcinogenesis (Weitzman and Gordon 1990). Although normal cells are able to efficiently repair the products of most promutagenic HOCl-mediated damage, no repair activity has been identified for 5-chlorocytosine, probably because 5-chlorocytosine mimics 5-methylcytosine (Lao et al. 2009). The presence of 5-chlorocytosine, which can be misrecognized by cellular machinery as 5-methylcytosine, would alter methylation patterns. In addition, 5-chlorocytosine is easily transformed to 5-chlorouracil (Theruvathu et al. 2009). Because 5-chlorouracil residues can pair with adenine as well as guanine (Kim et al. 2010), 5-chlorouracil–adenine base pairing might induce genetic mutation. Consequently, halogenated DNA is potentially able to induce both epigenetic and genetic changes that contribute to carcinogenesis.

Inoue et al. (1989) detected BT in human urine after benzene exposure; the urinary concentration of BT linearly correlated with the degree of benzene exposure, reaching > 50 mg/L (396 μM) in a worker exposed to 210 ppm benzene. In addition, Aksoy (1989) reported on Turkish workers who were chronically exposed to up to 650 ppm benzene, some of whom developed leukemia. We believe that it is plausible that 50 μM BT, the concentration used in the present study, could have been present in the workers.

Individuals with *MPO* polymorphism –463G→A in the promoter region, which reduces MPO expression, have decreased risk for various cancers (Cascorbi et al. 2000). Lan et al. (2004) reported that benzene-exposed workers with the –463G genotype showed greater hematotoxicity than did workers with the –463A genotype. These findings suggest important roles in myelotoxicity or carcinogenesis for MPO-catalyzed reactions toward HOCl.

**Figure 6:**
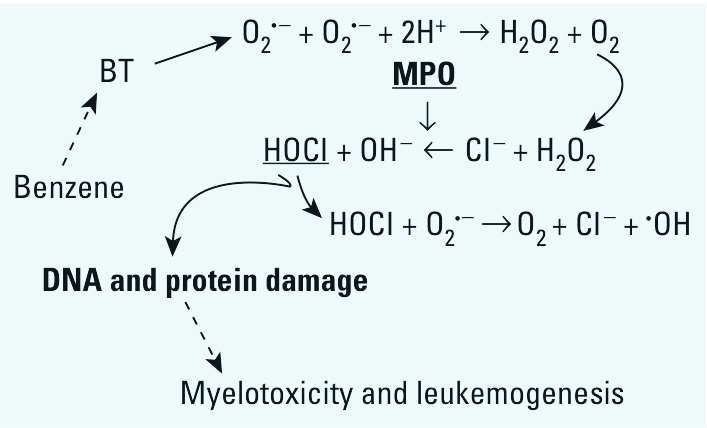
Hypothesized mechanism of cytotoxicity of BT involved in myelotoxicity and leukemogenesis of benzene.

In the present study we have constructed a novel hypothesis (Figure 6) that exposure to BT increases O_2_^•−^ generation, possibly by autoxidation. The O_2_^•−^ is chemically or enzymatically converted to H_2_O_2_, which is then metabolized to HOCl by MPO; this HOCl halogenates DNA and proteins, thus inducing myelotoxicity or leukemogenesis. The high expression of MPO from myeloid cells, along with the fact that halogenated DNA can cause gene mutation and epigenetic changes, may explain how benzene is involved in bone marrow disorders or myeloid leukemia. A previous study of benzene toxicity reported that MPO plays a role in the bioactivation of benzene’s phenolic metabolites (Eastmond et al. 2005). Here, we show for the first time that a benzene metabolite, BT, is capable of generating HOCl and consequent halogenative damage via the H_2_O_2_–MPO–HOCl system. Our findings lend strong support to the hypothesis that BT-induced DNA halogenation is a primary reaction in leukemogenesis associated with benzene.

## References

[r1] Aksoy M. (1989). Hematotoxicity and carcinogenicity of benzene.. Environ Health Perspect.

[r2] Cascorbi I, Henning S, Brockmöller J, Gephart J, Meisel C, Müller JM (2000). Substantially reduced risk of cancer of the aerodigestive tract in subjects with variant –463A of the myeloperoxidase gene.. Cancer Res.

[r3] Eastmond DA, Mondrala ST, Hasegawa L (2005). Topoisomerase II inhibition by myeloperoxidase-activated hydroquinone: a potential mechanism underlying the genotoxic and carcinogenic effects of benzene.. Chem Biol Interact.

[r4] Gujral JS, Farhood A, Bajt ML, Jaeschke H (2003). Neutrophils aggravate acute liver injury during obstructive cholestasis in bile duct-ligated mice.. Hepatology.

[r5] Heller JI, Crowley JR, Hazen SL, Salvay DM, Wagner P, Pennathur S (2000). *p*-Hydroxyphenylacetaldehyde, an aldehyde generated by myeloperoxidase, modifies phospholipid amino groups of low density lipoprotein in human atherosclerotic intima.. J Biol Chem.

[r6] Henderson JP, Byun J, Heinecke JW (1999). Molecular chlorine generated by the myeloperoxidase-hydrogen peroxide-chloride system of phagocytes produces 5-chlorocytosine in bacterial RNA.. J Biol Chem.

[r7] Henderson RF, Sabourin PJ, Bechtold WE, Griffith WC, Medinsky MA, Birnbaum LS (1989). The effect of dose, dose rate, route of administration, and species on tissue and blood levels of benzene metabolites.. Environ Health Perspect.

[r8] Huff J. (2007). Benzene-induced cancers: abridged history and occupational health impact.. Int J Occup Environ Health.

[r9] Hurst JK, Barrette WC (1989). Leukocytic oxygen activation and microbicidal oxidative toxins.. Crit Rev Biochem Mol Biol.

[r10] IARC (International Agency for Research on Cancer) (2011). Agents Classified by the *IARC Monographs*, Volumes 1–102.. http://monographs.iarc.fr/ENG/Classification/ClassificationsAlphaOrder.pdf.

[r11] Inoue O, Seiji K, Nakatsuka H, Watanabe T, Yin S, Li GL (1989). Excretion of 1,2,4-benzenetriol in the urine of workers exposed to benzene.. Br J Ind Med.

[r12] Kato Y, Kawai Y, Morinaga H, Kondo H, Dozaki N, Kitamoto N (2005). Immunogenicity of a brominated protein and successive establishment of a monoclonal antibody to dihalogenated tyrosine.. Free Radic Biol Med.

[r13] Kawai Y, Matsui Y, Kondo H, Morinaga H, Uchida K, Miyoshi N (2008). Galloylated catechins as potent inhibitors of hypochlorous acid-induced DNA damage.. Chem Res Toxicol.

[r14] Kawai Y, Morinaga H, Kondo H, Miyoshi N, Nakamura Y, Uchida K (2004). Endogeneous formation of novel halogenated 2’-deoxycytidine. Hypohalous acid-mediated DNA modification at the site of inflammation.. J Biol Chem.

[r15] Kawanishi S, Inoue S, Kawanishi M. (1989). Human DNA damage induced by 1,2,4-benzenetriol, a benzene metabolite.. Cancer Res.

[r16] Kim CH, Darwanto A, Theruvathu JA, Herring JL, Sowers LC (2010). Polymerase incorporation and miscoding properties of 5-chlorouracil.. Chem Res Toxicol.

[r17] Kohanski MA, Dwyer DJ, Hayete B, Lawrence CA, Collins JJ (2007). A common mechanism of cellular death induced by bacterial antibiotics.. Cell.

[r18] Kolachana P, Subrahmanyam VV, Meyer KB, Zhang L, Smith MT (1993). Benzene and its phenolic metabolites produce oxidative DNA damage in HL60 cells *in vitro* and in the bone marrow *in vivo*.. Cancer Res.

[r19] Lan Q, Zhang L, Li G, Vermeulen R, Weinberg RS, Dosemeci M (2004). Hematotoxicity in workers exposed to low levels of benzene.. Science.

[r20] Lao VV, Herring JL, Kim CH, Darwanto A, Soto U, Sowers LC (2009). Incorporation of 5-chlorocytosine into mammalian DNA results in heritable gene silencing and altered cytosine methylation patterns.. Carcinogenesis.

[r21] Levitt J. (2007). The safety of hydroquinone: a dermatologist’s response to the 2006 *Federal Register*.. J Am Acad Dermatol.

[r22] Lewis JG, Stewart W, Adams DO (1988). Role of oxygen radicals in induction of DNA damage by metabolites of benzene.. Cancer Res.

[r23] Marnett LJ (2000). Oxyradicals and DNA damage.. Carcinogenesis.

[r24] Matsugo S, Sasai M, Shinmori H, Yasui F, Takeuchi M, Takeuchi T. (2006). Generation of a novel fluorescent product, monochlorofluorescein from dichlorofluorescin by photo-irradiation.. Free Radic Res.

[r25] Nakazato T, Sagawa M, Yamato K, Xian M, Yamamoto T, Suematsu M (2007). Myeloperoxidase is a key regulator of oxidative stress-mediated apoptosis in myeloid leukemic cells.. Clin Cancer Res.

[r26] Ohnishi S, Murata M, Kawanishi S. (2002). DNA damage induced by hypochlorite and hypobromite with reference to inflammation-associated carcinogenesis.. Cancer Lett.

[r27] Oikawa S, Hirosawa I, Hirakawa K, Kawanishi S. (2001). Site specificity and mechanism of oxidative DNA damage induced by carcinogenic catechol.. Carcinogenesis.

[r28] Rothe G, Valet G. (1990). Flow cytometric analysis of respiratory burst activity in phagocytes with hydroethidine and 2’,7’-dichlorofluorescin.. J Leukoc Biol.

[r29] Setsukinai K, Urano Y, Kakinuma K, Majima HJ, Nagano T (2003). Development of novel fluorescence probes that can reliably detect reactive oxygen species and distinguish specific species.. J Biol Chem.

[r30] Snyder R, Hedli CC (1996). An overview of benzene metabolism.. Environ Health Perspect.

[r31] Takeuchi T, Nakajima M, Morimoto K. (1996). Relationship between the intracellular reactive oxygen species and the induction of oxidative DNA damage in human neutrophil-like cells.. Carcinogenesis.

[r32] Takeuchi T, Nakajima M, Ohta Y, Mure K, Takeshita T, Morimoto K. (1994). Evaluation of 8-hydroxydeoxyguanosine, a typical oxidative DNA damage, in human leukocytes.. Carcinogenesis.

[r33] Theruvathu JA, Kim CH, Darwanto A, Neidigh JW, Sowers LC (2009). pH-Dependent configurations of a 5-chlorouracil-guanine base pair.. Biochemistry.

[r34] Tomono S, Miyoshi N, Sato K, Ohba Y, Ohshima H. (2009). Formation of cholesterol ozonolysis products through an ozone-free mechanism mediated by the myeloperoxidase-H_2_O_2_-chloride system.. Biochem Biophys Res Commun.

[r35] Weitzman SA, Gordon LI (1990). Inflammation and cancer: role of phagocyte-generated oxidants in carcinogenesis.. Blood.

[r36] Whiteman M, Jenner A, Halliwell B. (1997). Hypochlorous acid-induced base modifications in isolated calf thymus DNA.. Chem Res Toxicol.

[r37] Whysner J, Reddy MV, Ross PM, Mohan M, Lax EA (2004). Genotoxicity of benzene and its metabolites.. Mutat Res.

[r38] Zhang L, Bandy B, Davison AJ (1996). Effects of metals, ligands and antioxidants on the reaction of oxygen with 1,2,4-benzenetriol.. Free Radic Biol Med.

